# TRIP13 promotes tumor growth and is associated with poor prognosis in colorectal cancer

**DOI:** 10.1038/s41419-018-0434-z

**Published:** 2018-03-14

**Authors:** Nengquan Sheng, Li Yan, Kai Wu, Weiqiang You, Jianfeng Gong, Landian Hu, Gewen Tan, Hongqi Chen, Zhigang Wang

**Affiliations:** 1 Department of General Surgery, Shanghai Jiao Tong University Affiliated Sixth People’s Hospital, Shanghai, 200233 China; 2 State Key Laboratory of Medical Genomics, Shanghai Institutes for Biological Sciences, Chinese Academy of Sciences, Shanghai, 200031 China

## Abstract

Colorectal cancer (CRC) is one of the most common neoplasms worldwide. However, the mechanisms underlying its development are still poorly understood. Thyroid hormone Receptor Interactor 13 (TRIP13) is a key mitosis regulator, and recent evidence has shown that it is an oncogene. Here, we report that TRIP13, which is overexpressed in CRC, is correlated with the CEA (carcino-embryonic antigen), CA19-9 (carbohydrate antigen 19-9) and pTNM (pathologic primary tumor, lymph nodes, distant metastasis) classification. Multivariate analyses showed that TRIP13 might serve as an independent prognostic marker of CRC. We also found that TRIP13 promoted CRC cell proliferation, invasion and migration in vitro and subcutaneous tumor formation in vivo. Furthermore, the potential mechanism underlying these effects involves the interaction of TRIP13 with a 14-3-3 protein, YWHAZ, which mediates G2-M transition and epithelial-mesenchymal transition (EMT). Together, these findings suggest that TRIP13 may be a potential biomarker and therapeutic target for CRC.

## Introduction

Colorectal cancer (CRC) is one of the most prevalent and fatal malignancies worldwide^1^. In China, CRC is among the top five cancers both in new diagnoses and in the leading cause of death^2^. Although great progress in surgery and chemotherapy has been achieved during the past few decades, the 5-year overall survival rate for stage III CRC is 59.5% and only 8.1% for stage IV CRC patients^3^. Relapse and metastasis are the main causes of high mortality, but the underlying molecular mechanisms have not been fully elucidated.

Thyroid hormone Receptor Interactor 13 (TRIP13), has been found to play a key role in meiotic recombination, spindle assembly checkpoint and chromosome synapsis^4^. Previous reports have indicated that TRIP13 was overexpressed in multiple neoplasms^5–7^. Recently, one study from Japan indicated that TRIP13 may act as an oncogene in colorectal cancer cells, but the mechanism was not clarified^8^. In this study, we used multiple methods to demonstrate that TRIP13 can promote CRC cell proliferation, migration, and invasion in vitro and subcutaneous implanted tumor formation in vivo, and it predicted poor survival of CRC patients. The potential mechanism underlying these effects involves TRIP13 interaction with YWHAZ, a member of the 14-3-3 protein family, which mediates G2-M transition and epithelial-mesenchymal transition (EMT).

## Results

### Expression of TRIP13 in human CRC tissues

**Fig. 1:**
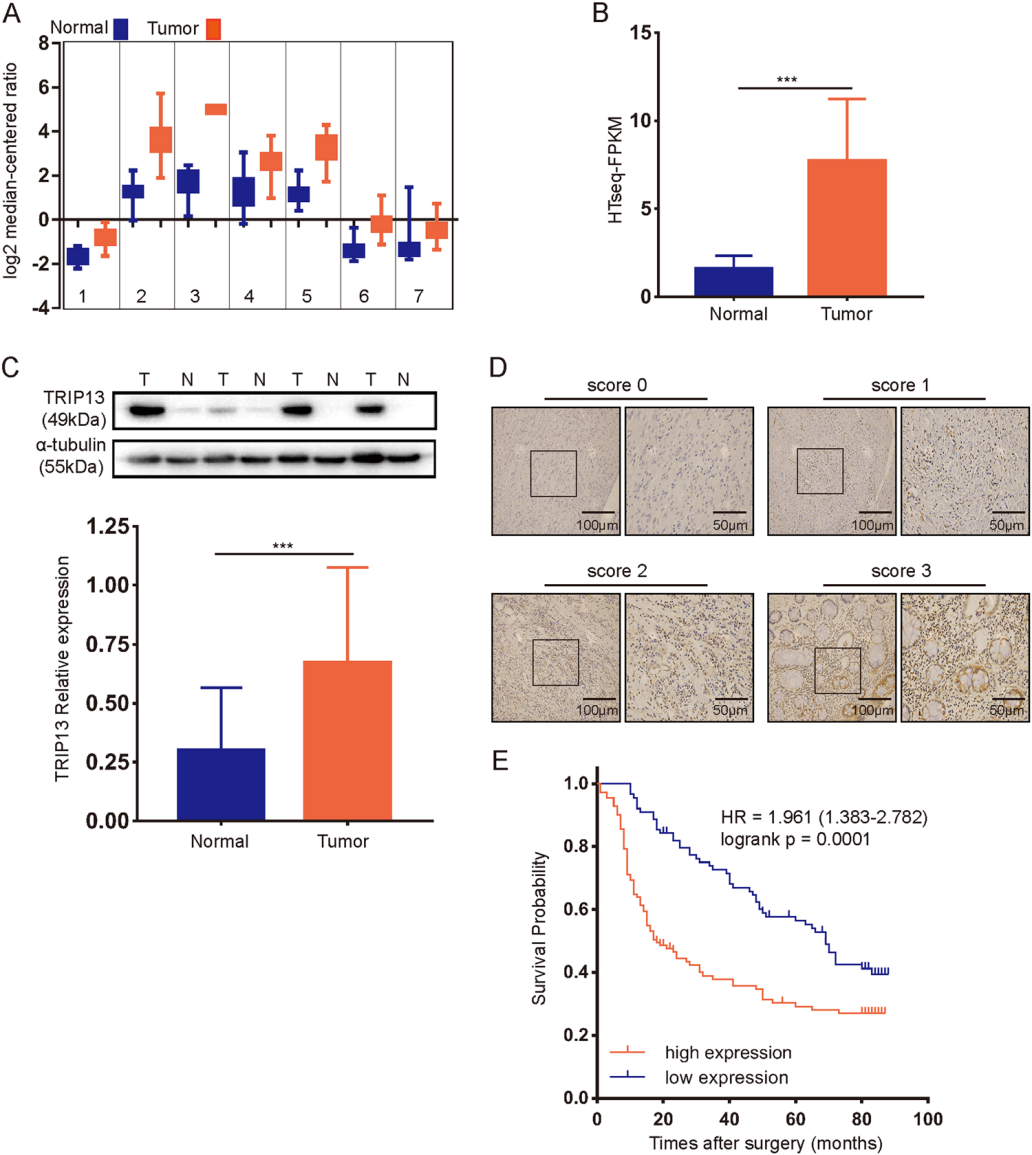
TRIP13 is overexpressed in CRC and associated with poor prognosis. **a** Data set from Oncomine showing that TRIP13 is upregulated in tumor tissue compared to normal tissue. **b** TRIP13 mRNA is overexpressed in tumor tissue compared with normal tissue in 41 paired CRC patients from TCGA. *n* = 41, *p* < 0.001. **c** Western blotting of 45 paired tumor and adjacent normal tissues shows that TRIP13 is highly expressed in tumor tissue. The upper panel is the signal intensity of TRIP13 and GAPDH; the lower panel shows the quantified results. *n* = 45, *p* < 0.001. **d** TRIP13 IHC scores of the staining intensity level in representative tumor tissues. The bars are as indicated. **e** OS was compared between patients with high and low expression of TRIP13. *p* = 0.0001

To determine the expression profile of TRIP13 in CRC, we analyzed multiple CRC data sets available from Oncomine, and TRIP13 was found to be upregulated in tumor tissue compared with normal tissue in these data sets (Fig. 1a from left to right: Graudens Colon^9^, Hong colorectal^10^, Skrzypczak colorectal2^11^, Skrzypczak colorectal^11^, Sabates Bellver colon^12^, Gaedcke colorectal^13^, Ki colon^14^.) The accession numbers are GSE3964, GSE9348, GSE20916, GSE20196, GSE8671, GSE20842, and GSE6988. To validate the results, we examined the mRNA level of TRIP13 in 41 pairs of CRC and corresponding normal tissues from TCGA (The Cancer Genome Atlas) data set. The results also showed that TRIP13 was highly expressed in tumor tissue (Fig. 1b, *p* < 0.001).

Furthermore, to assess the protein level of TRIP13 in CRC, western blotting was performed in 45 paired CRC and adjacent normal tissues, and we found that TRIP13 was significantly overexpressed in tumors compared with the matched normal tissues (Fig. 1c, *p* < 0.001). Together, these data indicate that TRIP13 is upregulated in CRC.

### Increased TRIP13 expression contributes to poor prognosis in CRC patients

To investigate the potential prognostic value of TRIP13 in CRC, immunohistochemistry was performed with 200 CRC formalin-fixed, paraffin-embedded (FFPE) tumor slides with complete clinicopathological characteristics and follow-up data. These patients were divided into high and low groups according to TRIP13 expression using the scoring system described in the Methods (Fig. 1d). We found that high TRIP13 expression was significantly associated with advanced pTNM stage and higher CEA and CA19-9 expression (Table 1).

**Table 1:** Comparison of clinicopathological profiles between low and high TRIP13 expression in CRC patients

	Low expression (*n* = 89)		High expression (*n* = 111)		*p*
	*n*	%	*n*	%	
*Age*					0.294
<60	31	34.8	31	27.9	
≥60	58	65.2	80	72.1	
*Gender*					0.938
Female	38	42.7	48	43.2	
Male	51	57.3	63	56.8	
*Tumor location*					0.709
Rectal	27	30.3	31	27.9	
Colon	62	69.7	80	72.1	
*Tumor size*					0.884
<15 cm^3^	28	31.5	36	32.4	
≥15 cm^3^	61	68.5	75	67.6	
*Differentiation*					0.49
Poor	18	20.2	27	24.3	
Moderate & well	71	79.8	84	75.7	
*pTNM*					0.005**
I-II	49	55.1	39	35.1	
III-IV	40	44.9	72	64.9	
*CEA*					0.045*
<5 μg/l	59	66.3	58	52.3	
≥5 μg/l	30	33.7	53	47.7	
*CA125*					0.378
<35 U/ml	66	74.2	76	68.5	
≥35 U/ml	23	25.8	35	31.5	
*CA19-9*					0.043*
<37 U/ml	68	76.4	70	63.1	
≥37 U/ml	21	23.6	41	36.9	

Chi-square test was performed, and *p* < 0.05 was considered statistically significantly. *p < 0.05; **p < 0.01

*pTNM* pathological tumor, lymph node, metastasis classification, *CEA* carcino-embryonic antigen, *CA125* carbohydrate antigen-125, *CA19-9* carbohydrate antigen-19-9

In addition, Cox proportional hazards regression analyses (Table 2) demonstrated that TRIP13 was an independent prognostic predictor for overall survival (HR = 1.887, *p* < 0.001). Moreover, Kaplan-Meier analysis showed that patients with high TRIP13 expression exhibited poorer survival than those with low expression (Fig. 1e, HR = 1.961, *p* = 0.0001). These data indicated that TRIP13 is significantly associated with CRC patient prognosis.

**Table 2:** Univariate and multivariate analysis of factors associated with overall survival in CRC patients

Features	Overall survival			
	Univariate	Multivariate		
	*p*-value	HR	95% CI	*p*-value
Age: ≥60 vs. <60 years	0.295			NA
Gender: male vs. female	0.276			NA
Tumor location: colon vs. rectal	0.394			NA
Differentiation: poor vs. moderate & well	0.882			NA
Tumor size: ≥15 vs. <15 cm^3^	0.063			NA
pTNM: I–II vs. III–IV	<0.001***	3.391	2.291–5.019	<0.001***
CEA: ≥5 vs. <5 ug/l	<0.001***	1.7	1.2–2.406	0.03*
CA125: ≥35 vs. <35 U/ml	0.153			NA
CA19-9: ≥37 vs. <37 U/ml	0.003**			NS
TRIP13: high vs. low	<0.001***	1.887	1.321–2.696	<0.001***

*NA* not adopted, *NS* not significant. *p < 0.05; **p < 0.01; ***p < 0.001

### Altered TRIP13 in CRC cells and its effect on tumorigenic properties in vitro

**Fig. 2:**
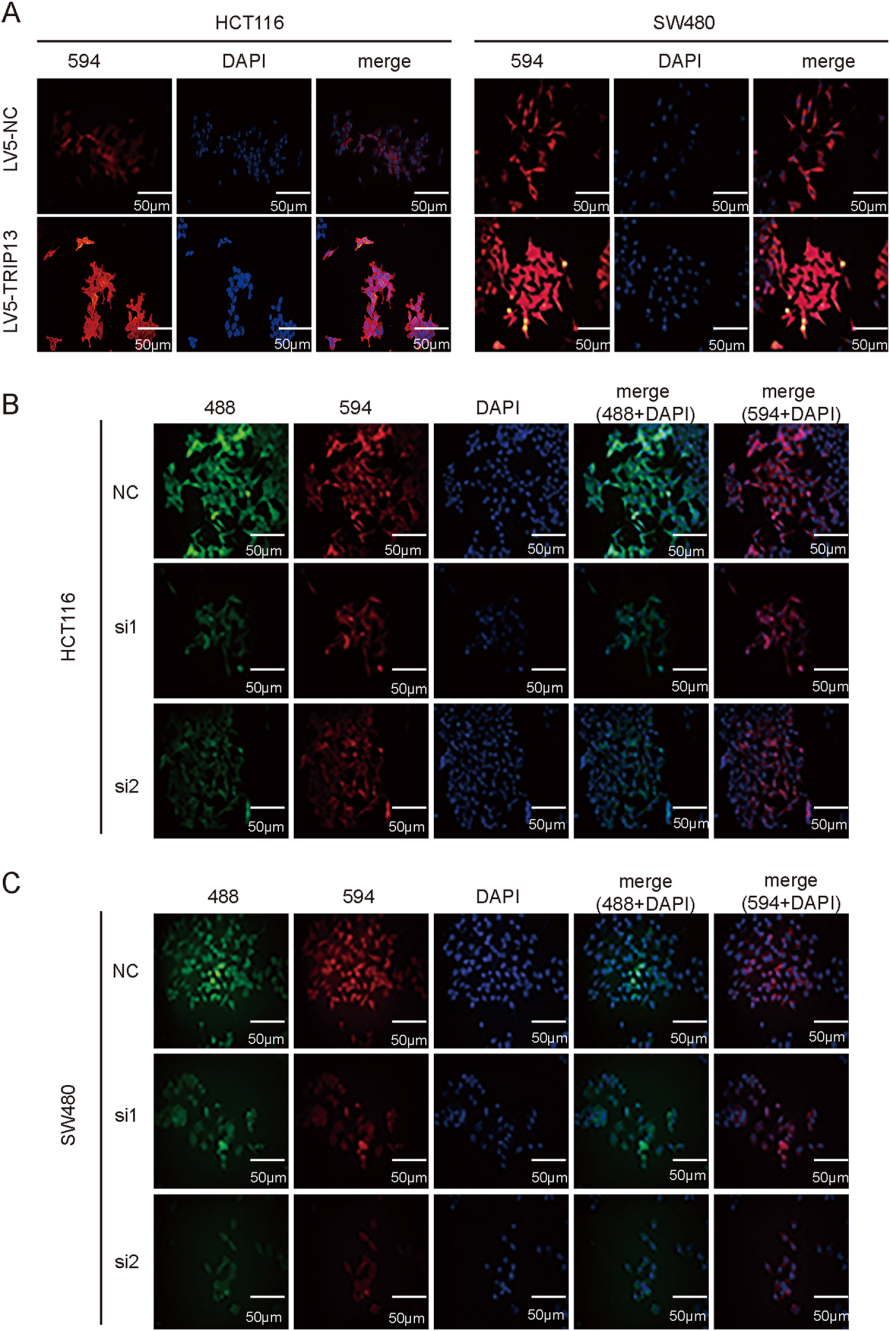
Transfection efficiency was examined with an immunofluorescence assay. **a** IF was performed to validate the overexpression efficiency of TRIP13 in HCT116 and SW480 cells. Red indicates TRIP13, and blue indicates cell nuclei. The bar is 50 μm. **b** and** c** IF was performed to validate the knockdown efficiency of TRIP13 in SW480 and HCT116 cells. Red and green indicate TRIP13, and blue indicates cell nuclei. The bar is 50 μm

**Fig. 3:**
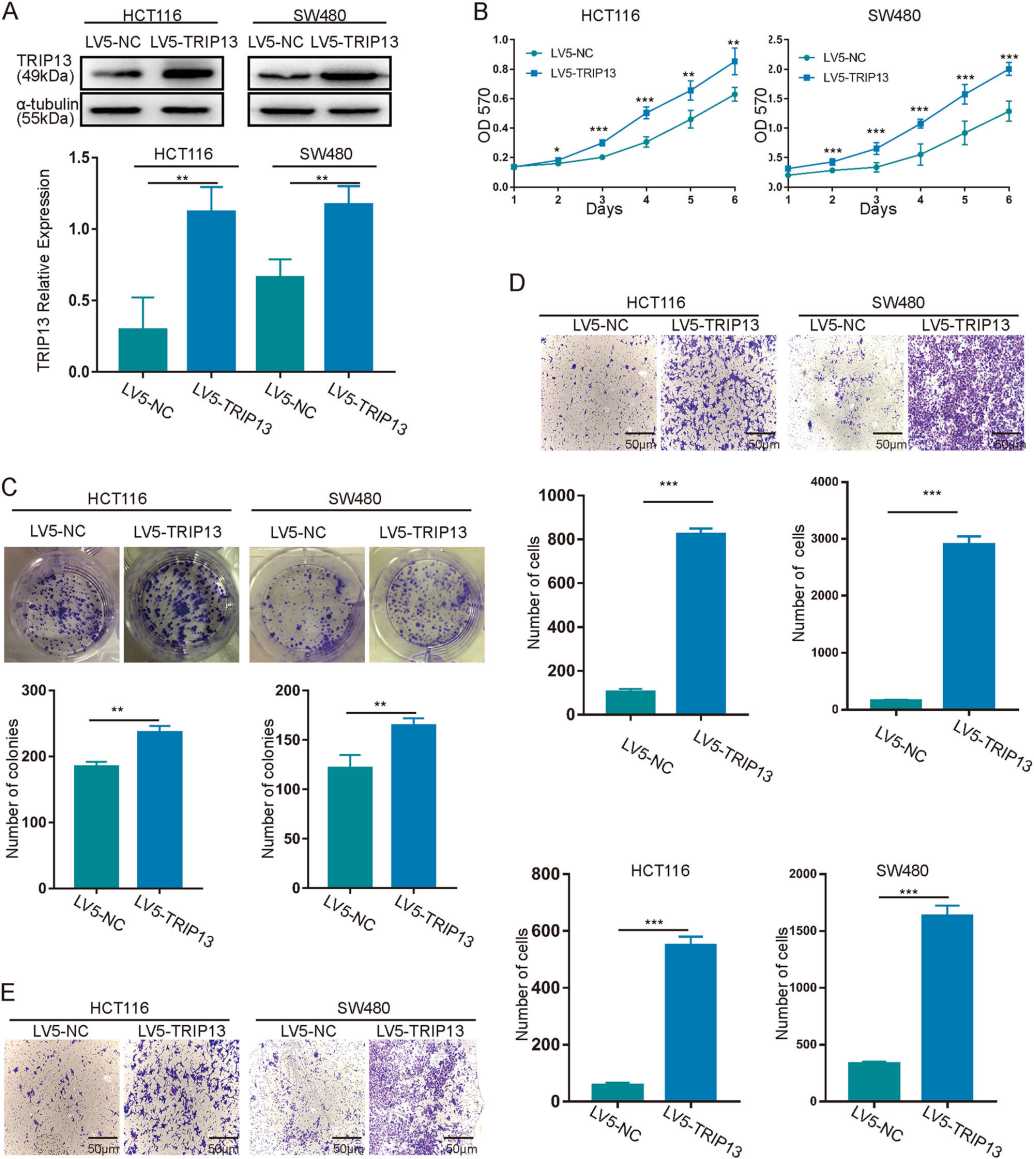
Upregulation of TRIP13 promotes tumorigenic properties in vitro. **a** Validation of the efficiency of TRIP13 expression. The upper panel is signal intensity; the lower panel is the quantified results. The values indicate the mean ± standard deviation. *p* < 0.01. **b**–**e** The effects of TRIP13 gain of function on in vitro proliferation (**b**, **c**), migration (**d**), and invasion (**e**). TRIP13 up-regulation significantly increased cell proliferation, migration and invasion abilities. The values indicate the mean ± standard deviation. *p < 0.05; **p < 0.01; ***p < 0.001

**Fig. 4:**
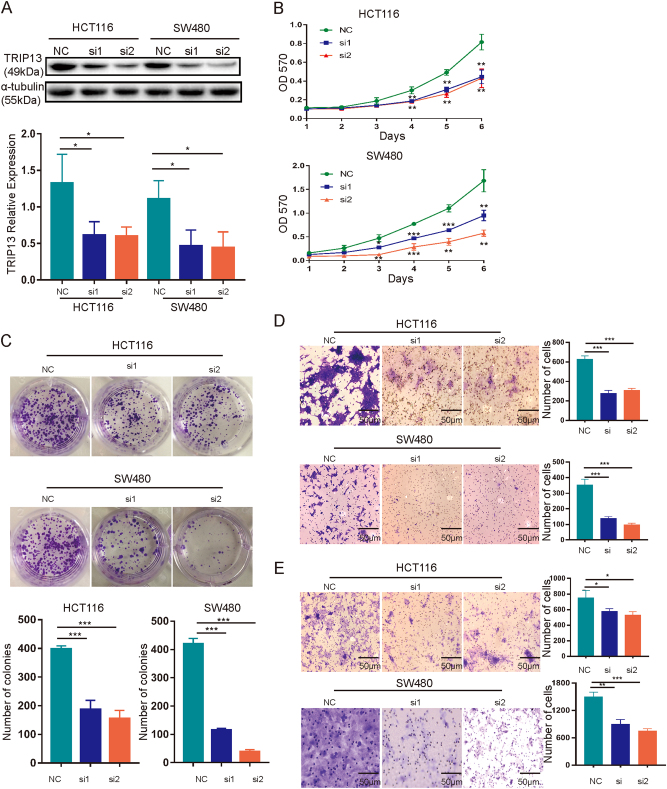
Suppression of TRIP13 inhibits the oncogenic phenotype in vitro. **a** Confirmation of the efficiency of TRIP13 knockdown. The left panel is signal intensity, and the right panel is the quantified results. The values indicate the mean ± standard deviation. *p* < 0.05. **b**–**e** The effects of TRIP13 loss of function on in vitro proliferation (**b**, **c**), migration (**d**), and invasion (**e**). TRIP13 knockdown inhibits cell proliferation, migration and invasion abilities. The values indicate the mean ± standard deviation. *p < 0.05; **p < 0.01; ***p < 0.001

To evaluate the biology function of TRIP13 in CRC cells, TRIP13 expression was knocked down with siRNAs and LV10-shRNA, and TRIP13 overexpression was induced with LV5-TRIP13. The transfection efficiency and TRIP13 protein expression level were validated by immunofluorescence and western blotting, respectively (Fig. 2a and Fig. 3a for TRIP13 overexpression, Fig. 2b–c and Fig. 4a for TRIP13 knockdown). (Fig. 2a and Fig. 3a for TRIP13 overexpression, Figs. 2b, c and Fig. 4a for TRIP13 knockdown). Simultaneously, we cloned the second siRNA sequence (si2) that targeted TRIP13 into an LV10 vector that also encoded RFP.

Then, we examined the effect of TRIP13 overexpression and knockdown on the proliferative ability of cells using MTT and colony formation assays. The results showed that overexpression of TRIP13 increased cell viability compared with the negative control (Fig. 3b), and this promotive effect was validated by the colony formation assay, in which TRIP13 led to increased colony formation (Fig. 3c). And knockdown of TRIP13 decreased cell viability (Figs. 4b, c). Furthermore, transwell migration and invasion assays were employed to assess the influence of TRIP13 on cell migration and invasion. These assays showed that cells overexpressing TRIP13 have increased migration and invasion ability compared with negative control cells (Figs. 3d, e), while knockdown TRIP13 induced the opposite effect (Figs. 4d, e). In addition, similar results with HCT116 and SW480 cells transduced with LV10-NC and LV10-shTRIP13 (Supplementary Figure 1A-D). Taken together, these data indicate that TRIP13 promotes the proliferation, invasion and migration ability of CRC cells.

### Altered TRIP13 expression affects tumorigenicity in vivo

**Fig. 5:**
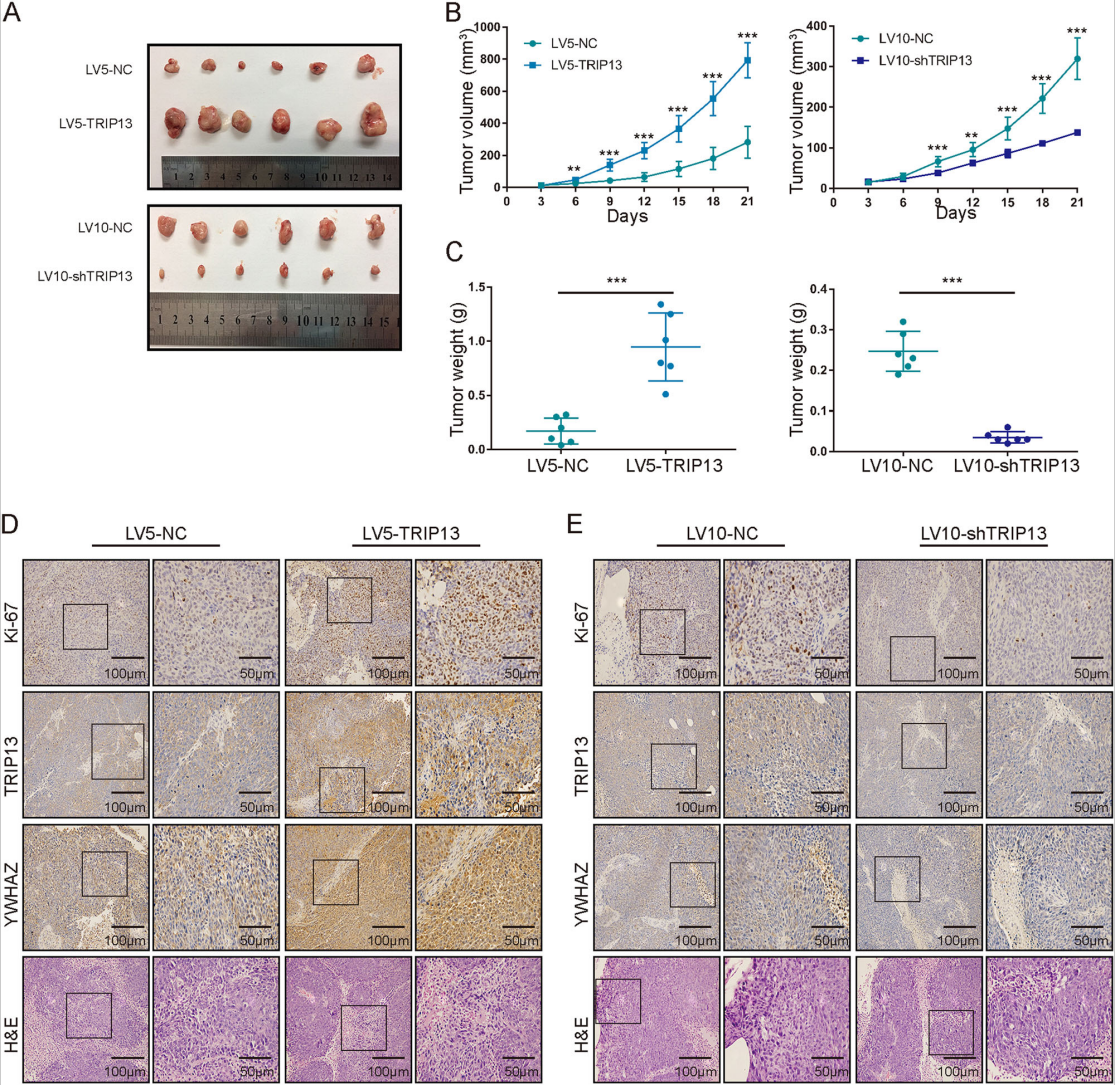
Altered TRIP13 expression affects tumorigenicity in vivo. **a** Tumors in nude mice bearing HCT116 cells in different groups. The upper panel is TRIP13 overexpression and the negative control; the lower panel is TRIP13 knockdown and the negative control. Each group contains six mice. **b** Statistical analysis of tumor volume in different groups. The left panel shows the overexpression and control groups, and the right panel shows the knockdown and control groups. The values indicate the mean ± standard deviation. *n* = 6/group. **p < 0.01; ***p < 0.001. **c** Statistical analysis of tumor weight in different groups. The left panel shows the overexpression and control groups, and the right panel shows the knockdown and control groups. The values indicate the mean ± standard deviation. *n* = 6/group. ***p < 0.001. **d** Representative staining images of TRIP13 overexpressing tumors. The bars are as indicated. **e** Representative staining images of TRIP13 knockdown tumors. The bars are as indicated

The above studies demonstrated that TRIP13 promotes the tumorigenic potential of CRC in vitro. Next, we explored the effect of TRIP13 on tumorigenesis in vivo using a xenograft model. Nude mice injected with LV5-TRIP13-infected HCT116 cells were found to have much larger tumors than those injected with LV5-NC-transfected HCT116 cells (Fig. 5a, top panel; Fig. 5b, left panel). Knockdown of TRIP13 had the opposite effect, and the subcutaneous tumors formed from LV10-shTRIP13-infected HCT116 cells were smaller than those formed from LV10-NC-infected HCT116 cells (Fig. 5a, bottom panel; Fig. 5b, right panel). These results were validated by the tumor weights (Fig. 5c).

Then, H&E staining was used to confirm the tumorigenic characteristics of these subcutaneous tumors (Figs. 5d, e, bottom panel), and immunohistochemistry staining was performed with those confirmed cancer tissues. The results showed that TRIP13 was high expressed in TRIP13 overexpressing tumors and low expressed in TRIP13 knockdown tumors (Figs. 5d, e, the second panel). In addition, Ki-67 immunohistochemistry staining was used to assess the proliferative index of these tumors. The results showed that upregulation of TRIP13 increased the proliferative ability of cells, but downregulation of TRIP13 suppressed the cell proliferative ability (Figs. 5d, e, the first panel). Together, these data suggest that TRIP13 may promote tumorigenesis in vivo.

### TRIP13 is correlated with EMT in CRC cells

**Fig. 6:**
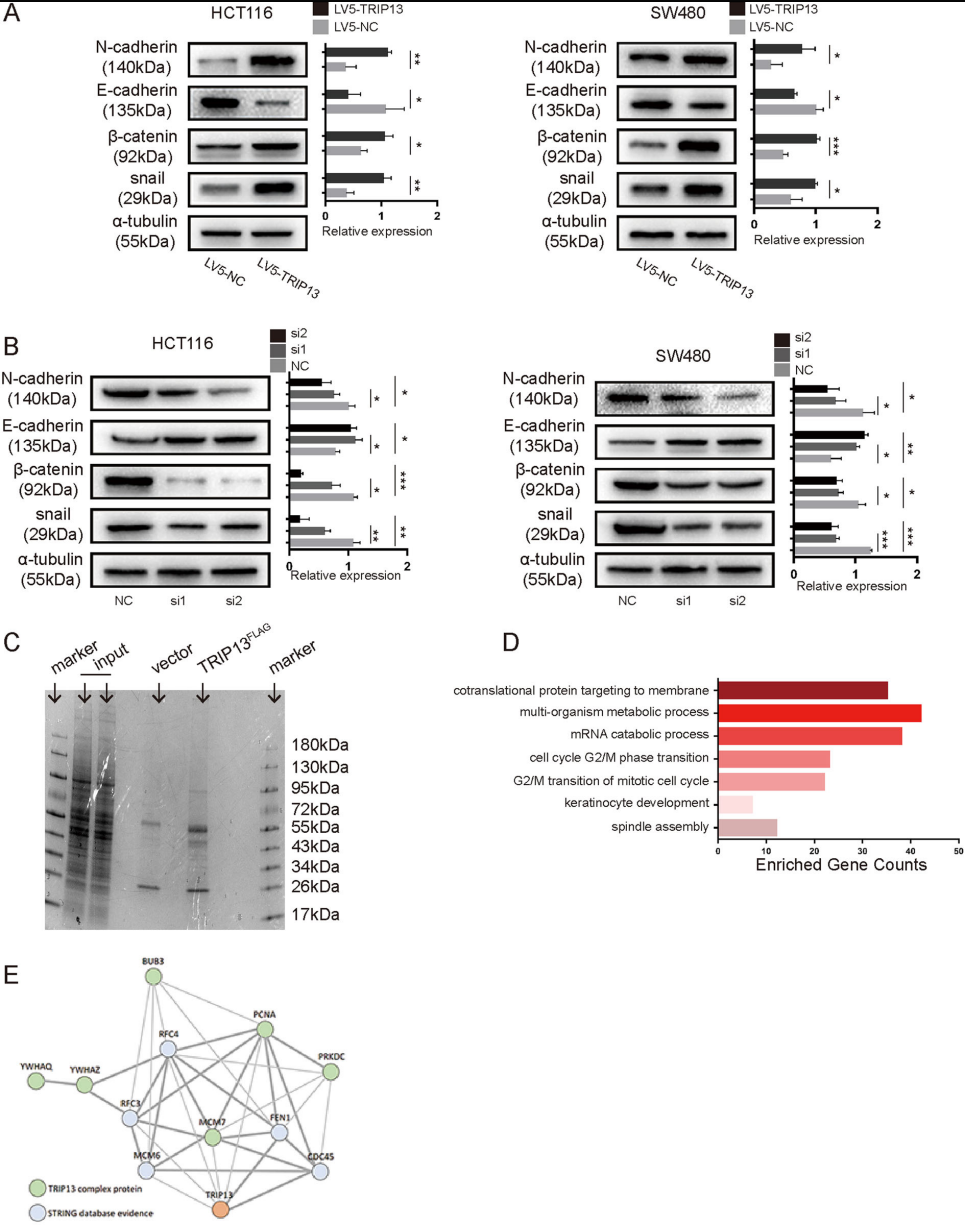
TRIP13 regulates G2-M transition and EMT. **a**, **b** Western blotting was performed in HCT116 and SW480 cells to determine the change in EMT markers expression upon gain/loss function of TRIP13. The signal intensity and quantitative analysis are as shown. The values indicate the mean ± standard deviation. *p < 0.05; **p < 0.01; ***p < 0.001. **c** MS analysis of TRIP13-associated proteins. Cell lysate extract from Flag-tagged TRIP13-expressing or control was subjected to IP, and then, the IP complex was subjected to MS analysis. **d** GO analysis was performed to determine the biological processes in which TRIP13-interacting genes are involved. **e** The interaction network of TRIP13-interacting genes from STRING database

EMT is recognized as an important process during carcinoma progression^15^. To determine whether TRIP13 has a critical role in the CRC EMT process, we examined changes in known molecular markers of EMT using western blotting in both HCT116 and SW480 cells. The results showed that upregulation of TRIP13 increased the expression of N-cadherin, β-catenin and snail but suppressed E-cadherin expression (Fig. 6a). In contrast, knockdown of TRIP13 suppressed N-cadherin, β-catenin and snail and increased E-cadherin expression (Fig. 6b). Cells transduced with LV10-NC and LV10-shTRIP13 showed similar results (Supplementary Figure 1E). These data demonstrated that TRIP13 may promote CRC progression by inducing EMT.

### TRIP13 interacts with 14-3-3 and modulates cell cycle

To explore the molecular mechanism of TRIP13 in CRC, we sought to identify potential TRIP13-interacting proteins. Flag-tagged TRIP13 was expressed in HCT116 cells, stained and immunoblotted, and then quantified using nano-LC-MS/MS (Fig. 6c). A large number of proteins were found to interact with TRIP13, and the genes encoding these proteins were annotated (Supplementary Excel 1). Then, gene-ontology (GO) Enrichment Analysis was performed to annotate the biological processes in which these genes are involved (Supplementary Excel 2). We found that these genes were enriched in cell cycle process (Fig. 6d, Supplementary Excel 2). Furthermore, Kyoto Encyclopedia of Genes and Genomes (KEGG) pathway analysis validated that these genes participated in cell cycle process (Supplementary Excel 3). In addition, STRING database shows a clear interaction network between TRIP13-interacting genes and other DNA replication proteins (Fig. 6e). The TRIP13-interacting genes YWHAQ and YWHAZ are members of the 14-3-3 superfamily. A previous study has demonstrated that 14-3-3 proteins play a key role in cell cycle, apoptosis, and EMT^16^. Collectively, these data provide strong evidence that TRIP13 plays an important part in cell cycle regulation pathways.

### TRIP13 modulates EMT in an YWHAZ-regulated way

**Fig. 7:**
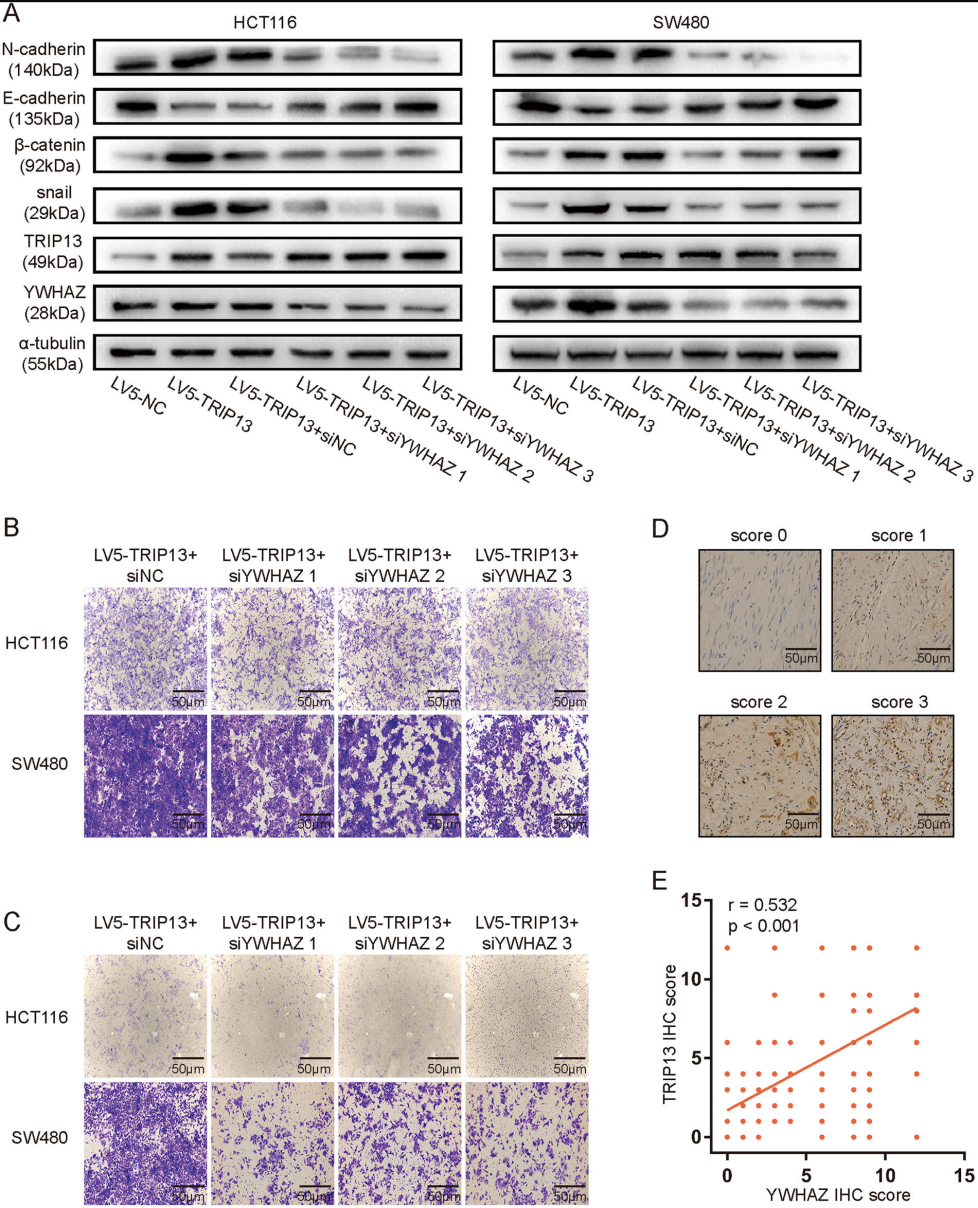
TRIP13 mediates EMT by interacting with YWHAZ. **a** Western blotting of EMT markers, TRIP13 and YWHAZ were performed in TRIP13 overexpressing CRC cells which transfected with siRNAs targeting YWHAZ, to determine whether TRIP13 mediates EMT in a YWHAZ dependent way. **b**, **c** The effect of knockdown of YWHAZ in TRIP13 overexpressing CRC cells on migration (**b**), and invasion (**c**). **d** YWHAZ IHC scores of the staining intensity level in representative tumor tissues. The bars are as indicated. **e** Spearman correlation coefficient plots for TRIP13 and YWHAZ. *r* = 0.532, 95%CI: (0.421–0.627), *p* < 0.001

As YWHAZ interacts with TRIP13 and mediates EMT, TRIP13 promotes EMT, we proposed that TRIP13 may mediate EMT dependent on interacting with YWHAZ. To assess this hypothesis, three different siRNAs (si1, si2, si3) targeting YWHAZ were applied to transfect TRIP13 overexpressing cells, the known molecular markers of EMT, TRIP13 and YWHAZ were examined by western blotting. The results showed that knockdown of YWHAZ neutralized the up-regulation effect of N-cadherin, β-catenin, snail, and the down-regulation effect of E-cadherin (Fig. 7a). And then down-regulation of YWHAZ induced inhibition of migration and invasion ability in both HCT116 and SW480 cells (Fig. 7b,c). Moreover, the expression of YWHAZ in xenograft tumors was examined by immunohistochemistry, the results showed that YWHAZ was high expressed in TRIP13 overexpressing tumors and low expressed in TRIP13 knockdown tumors (Figs. 5d, e, the third panel). Furthermore, we evaluated the expression of YWHAZ in the same 200 CRC FFPE tumor slices, the representative staining scores were shown in Fig. 7d. Spearman correlation analyses was conducted, and the result showed that TRIP13 and YWHAZ were positively related (Fig. 7e, *r* = 0.532, *p* < 0.001). These data together indicate that TRIP13 modulates EMT in an YWHAZ-dependent way.

## Discussion

In the past few decades, a series of biomarkers have been identified as having prognostic value and therapeutic significance in CRC. However, the overall survival of late stage CRC patients remains poor, and our understanding of the mechanisms of this disease is still inadequate. In this study, we demonstrated that TRIP13 is a potential CRC biomarker.

TRIP13, a member of the AAA + ATPase super-family, is located at 5p15.33 and encodes a protein with 432 amino acids. Studies have shown that TRIP13 is a key regulator of meiotic recombination and the spindle assembly checkpoint because TRIP13 catalyzes the conversion of C-Mad2 to inactive O-Mad2, with the help of adapter protein p31^comet^ to recruit TRIP13 to HORMA domain-closure motif complexes^17,18^. Despite playing a key role in meiotic regulation, overexpression or amplification of TRIP13 has been found in multiple human cancers^19,20^. In our study, TRIP13 was found to be highly expressed in CRC tissues compared with adjacent normal tissues, and this result was validated by the analysis of CRC data sets from Oncomine and TCGA. TRIP13 was first reported as an oncogene in 2014 in head and neck cancer, and several subsequent studies revealed that TRIP13 plays an oncogenic role in other neoplasms^5–8^. Kurita and his colleagues have demonstrated that TRIP13 involves in CRC cell proliferation and invasion and may be a potential target for CRC treatment^8^, but they only confirmed the oncogenic role of TRIP13 in vitro, and the mRNA level of TRIP13 was analyzed between normal and tumor tissues from unpaired patients. Here, we demonstrated that TRIP13 promotes HCT116 and SW480 cells proliferation, migration and invasion, as well as tumorigenicity in vivo. Furthermore, we analyzed TRIP13 with clinicopathological characteristics in 200 CRC patients, and found that TRIP13 was significantly associated with pTNM stage, CEA and CA19-9. pTNM can be considered as the gold standard for cancer treatment and prognostic predictor^21^, CEA and CA19-9 is used to detect relapse and follow response to therapy of CRC^22^. Moreover, multivariate analyses showed that TRIP13 can predict overall survival of CRC as well as pTNM and CEA. Therefore, TRIP13 may serve as a promising biomarker of CRC.

The mechanism by which TRIP13 contributes to tumors is still poorly understood. In multiple myeloma, researchers found that TRIP13 induces Mad2 degradation through the Akt pathway and abrogates spindle checkpoint^6^. As one of the mitotic checkpoint complexes, Mad2 is critical for chromosome segregation^23^. Degradation of Mad2 leads to chromosome mis-segregation during mitosis, which ultimately contributes to cancer development and chemotherapy resistance^24^. Zhou and his colleagues confirmed that the C-MYC/TRIP13/PUMA axis regulates chronic lymphocytic leukemia^7^. PUMA, a pro-apoptotic protein, was reported to be involved in p53-mediated apoptosis^25^. However, in this study, researchers found that PUMA mediates cell apoptosis in a TRIP13-dependent manner. As the first research indicated that TRIP13 acts as an oncogene, Banerjee and his colleagues utilized mass spectrometry and Co-IP to explore how TRIP13 promotes head and neck cancer progression, and they found that TRIP13 promotes non-homologous end joining (NHEJ) and induces chemoresistance^5^. Double-strand breaks are the most lethal type of DNA damage, and they are primarily repaired through homologous recombination (HR) or NHEJ^26^. NHEJ promotes cancer and chromosome instability because it is often inaccurate.

It is well recognized that EMT gives rise to the dissemination of tumor cells from primary sites, which polarized epithelial tumor cells acquired mesenchymal cell phenotype and enhanced migratory capacity and invasiveness^27^. So we examined the effect of TRIP13 on expression of critical EMT markers, E-cadherin, N-cadherin, β-catenin and snail, and found that TRIP13 promotes EMT process. In order to clarify the underlying mechanism, Mass spectrometry was performed to analyze proteins that interact with TRIP13, and then, GO and KEGG analysis were applied to explore the biological function and pathway of the genes encoding these proteins. The results indicated that TRIP13-interacting genes are involved in G2-M cell cycle transition. Cell cycle has four sequential phases, G1 (preparation for DNA synthesis), S (DNA replication), G2 (preparation for mitosis), and M (mitosis), and each phase is highly controlled and regulated by checkpoints, which aim to maintain genomic integrity by repairing damaged DNA before entering mitosis. If the damaged DNA cannot be repaired correctly, cell cycle will be arrested^28^. Checkpoints at G1-S transition and G2-M transition are the most important cell cycle checkpoints^28^. However, during cancer cell evolution, this mechanism is abolished due to mutated checkpoints, and then, damaged DNA can enter mitosis, which leads to uncontrolled proliferation and a malignant phenotype^29^. Our study demonstrated that TRIP13 interacts with YWHAQ and YWHAZ, which are members of the 14-3-3 protein superfamily. YWHAZ, also named 14-3-3ζ, plays key roles in cell cycle and EMT^16^, recent researches indicated that YWHAZ involves in tumor progression and could be a prognostic marker for kinds of cancers^30,31^. So we hypothesis that TRIP13 mediates EMT dependent on interacting with YWHAZ, knockdown of YWHAZ in TRIP13 overexpressing CRC cells and western blotting of E-cadherin, N-cadherin, β-catenin and snail were performed, the results showed that the up-regulation effect of N-cadherin, β-catenin, snail, and the down-regulation effect of E-cadherin induced by TRIP13 were rescued by YWHAZ knockdown, furthermore, we found that TRIP13 and YWHAZ were positively related. This data together confirmed our hypothesis, TRIP13 may promote CRC progression by interacting with YWHAZ to regulate EMT.

In sum, TRIP13 promotes CRC cell progression in vivo and in vitro and indicates poor CRC patient survival. The underlying mechanism involves regulation of G2-M transition and EMT through interacting with YWHAZ. Our study suggests that TRIP13 is a potential biomarker for CRC patients and might further assist in therapeutic decisions regarding CRC treatment.

## Materials and methods

### Data resource

The analyses of TRIP13 in CRC was performed using Oncomine (www.oncomine.org), which comprised of 7 groups. The number of tissues included was 12 normal and 18 tissues in group 1, 12 normal and 70 tumor tissues in group 2, 10 normal and 5 tumor tissues in group 3, 24 normal and 45 tumor tissues in group 4, 32 normal and 32 tumor tissues in group 5, 65 normal and 65 tumor tissues in group 6, 38 normal and 50 tumor tissues in group 7. We also downloaded level-3 RNAseq expression data of 41 tumor and normal paired COAD from TCGA database, the TRIP13 HTseq-FPKM in tumor and normal samples were abstracted and statistical analyses was used by t-test.

### Cell culture

The human CRC cell lines HCT116 and SW480 were purchased from Cell Bank of Chinese Academy of Sciences (Shanghai, China) and cultured in Dulbecco’s modified Eagle’s medium (DMEM) (Gibco, Carlsbad, USA) supplemented with 10% fetal bovine serum (FBS) (Gibco, Carlsbad, USA) and 1% penicillin/streptomycin (Gibco, Carlsbad, USA) in a humidified atmosphere of 5% CO_2_ at 37 °C.

### Tissue samples and clinicopathological information

CRC tissues and paired normal tissues were obtained from 45 patients who underwent surgical resection in the Department of General Surgery, Shanghai Jiao Tong University Affiliated Sixth People’s Hospital. The fresh samples were transported in liquid nitrogen and stored in −80 °C until protein extraction. FFPE tumor blocks from 200 CRC patients between January 2010 and January 2012 were obtained from the Department of Pathology. A complete follow-up was conducted every 6 months until July 2017. This study was approved by the Ethics Committee of Shanghai Jiao Tong University Affiliated Sixth People’s Hospital, and all patients provided informed consent.

### Immunohistochemistry staining and scoring

FFPE tumor blocks were cut into 5-μm-thick sections and mounted on Premiere microscope slides, and then, the slides were heated at 60 °C for 40 min. Deparaffinization and rehydration was performed as previously described ^32^. Antigen retrieval was performed with 10 nM citrate antigen retrieval solution (Sangon Biotech, Shanghai, China) at 95 °C for 10 min. After non-specific interactions were blocked with 3% goat serum, TRIP13 rabbit polyclonal antibody (Abcam, Cambridge, USA) was used at a dilution of 1:150, YWHAZ rabbit polyclonal antibody (Proteintech, Chicago, USA) was used at a dilution of 1:500, Ki-67 rabbit polyclonal antibody (Proteintech, Chicago, USA) with a dilution of 1:400, then incubated with the slides overnight at 4 °C. Five random fields in each slide were selected and evaluated independently by two pathologists. A semi-quantitative scoring system was used to evaluate the staining results basing on the percentage and intensity of positively stained cells. The intensity was scored as follows: 0-negative, 1-weak, 2-moderate, and 3-strong. The frequency of positively stained cells was defined as: 0-less than 5%, 1–5 to 25%, 2–26 to 50%, 3–51 to 75%, and 4-greater than 75%. For statistical analysis, scores of 0 to 7 were considered low expression, and scores of 8 to 12 were considered high expression.

### Western blot

Protein was prepared by lysing cells in radio immunoprecipitation buffer containing phosphatase and protease inhibitors (Beyotime Biotechnology, Nantong, China) and quantified using a BCA protein assay kit (Beyotime Biotechnology, Nantong, China). In brief, 50 µg of protein was resolved in 8–15% Tris-SDS-PAGE gels and transferred to PVDF membranes (Millipore, Billerica, USA). The membranes were probed with a primary anti-human TRIP13 rabbit antibody (Proteintech, Chicago, USA) at a dilution of 1:1000 and incubated with HRP-conjugated Affinipure goat anti-rabbit secondary antibody (Proteintech, Chicago, USA). Immunoreactive proteins were detected using ECL (Millipore, Billerica, USA) and a Bio-rad ChemoDoc MP System. The intensity of lanes was assessed using ImageJ (NIH, USA)

### Cell transfection and lentivirus infection

We successfully established stable TRIP13-overexpressing HCT116 and SW480 cell lines via infection with LV5-TRIP13 or LV5-NC lentivirus that also encoded GFP. And we constructed two different siRNAs (si1, si2) and then applied them to transfect HCT116 and SW480 cells using Lipofectamine 2000. Simultaneously, we cloned the second siRNA sequence (si2) that targeted TRIP13 into an LV10 vector that also encoded RFP. YWHAZ was knocked down with siRNAs. All of these constructs were purchased from GenePharma (Shanghai, China), and transduction was performed according to the specific manufacturer’s instructions.

### Cell viability and colony formation assay

Cell viability was assessed using an MTT Cell Proliferation and Cytotoxicity Detection Kit (KeyGEN BioTECH, Nanjing, China). Colony formation ability was assessed on 6 or 12-well plates for 10–14 days, and colonies were fixed with methanol and then stained with crystal violet.

### Transwell migration and invasion assay

Transwell assays were performed with 8-μm polycarbonate transwell filters (Coring, Cambridge, USA). Briefly, 3–5 × 10^5^ cells were seeded in the upper chamber without FBS, and the lower chamber was filled with 600 µl of DMEM containing 10% FBS. After 24–48 h of incubation, cells on the lower membrane were set in 4% paraformaldehyde and stained with crystal violet. Then, 5 random fields were selected and photographed.

### Immunofluorescence assay

After the indicated tumor cells crawled on the slide, treatmented with siRNA or lentivirus, the CRC cells were fixed in 4% paraformaldehyde for 15 min. Then, the slides were subjected to 0.2%Triton® X–100 (Sigma Aldrich, St. Louis, USA) for 20 min and washed with PBST. Blocking was carried out in 5% bovine serum, and then samples were incubated with anti-TRIP13 antibody (Proteintech, Chicago, USA) at a dilution of 1:50 overnight at 4 °C. Then, the cells were incubated with Alexa-labeled secondary antibodies (anti-rabbit IgG (H + L), F(ab’)2 fragment (Alexa Fluor® 488 Conjugate)/anti-rabbit IgG (H + L), F(ab’)2 fragment (Alexa Fluor® 594 Conjugate) 1:1000, Cell Signaling Technology, Danvers, MA, USA) for 30 min at room temperature. Nuclei were stained with DAPI (1:5000, Cell Signaling Technology, Danvers, USA). Following three final rinses with PBST, the cells were imaged with a fluorescence microscope (Olympus, Japan).

### Co-immunoprecipitation and peptide preparation

HCT116-Flag-TRIP13 cells were lysed (CST, Danvers, USA), and the protein was isolated. TRIP13 complex proteins were purified with anti-Flag M2 magnetic beads (Sigma-Aldrich, St. Louis, USA). The co-immunoprecipitation assay procedure was performed according to the manufacturer’s instructions. Then, the TRIP13 complex components were eluted in blue loading buffer (CST, Danvers, USA) and SDS-PAGE was performed (mini-PROTEAN precast gels, Bio-Rad). The protein lanes were visualized with Coomassie brilliant blue staining. Peptide preparation for mass spectrometric analysis was performed according to a previously described process^33^.

### Mass spectrometry and peptide data analysis

The prepared peptides were analyzed using nano-LC-MS/MS. LC separations were performed on an Easy nano LC system (Thermo Scientific, Bremen, Germany). Eluted peptides were directly analyzed using tandem mass spectrometry (MS/MS) on an LTQ-Orbitrap Velos Pro mass spectrometer (Thermo Scientific, Bremen, Germany) equipped with a nano-electrospray ion source. Proteome Discoverer 2.0 (Thermo Scientific) software was used to analyze the raw Xcalibur file generated from the mass spectrometer. The SEQUEST search algorithm was used to search unique peptides against a composite database comprising all the UniProt Homo sapiens databases (10/2016, 34361 entries). The minimum cutoff for peptide length was set at 7 amino acids, and the maximum permissible missed cleavage was set at 2. The positive proteins were identified with a local FDR < 1% and at least one unique peptide per protein.

### Tumor formation in nude mice

Female BALB/c nude mice (5 weeks old) were bred in a specific pathogen-free animal faculty and monitored every day. Human colon cancer HCT116 cells transduced with LV5-TRIP13 or LV5-negative control and LV10-shTRIP13 or LV10-negative control were suspended in serum-free DMEM (Gibco, Carlsbad, USA), and a total of 5 × 10^6^ cells were injected subcutaneously into the right flank of mice. The length and width of the resulting tumors were measured every 3 days starting 6 days after injection. Tumor volume was calculated using the formula (length × width^2^)/2. After 3 weeks, mice were sacrificed, and the tumors were collected and weighed. The experimental procedures were approved by the Ethical Committee of Shanghai Jiao Tong University Affiliated Sixth People’s Hospital.

### Statistical analysis

The data are presented as the mean ± standard deviation of at least three independent experiments. Difference between variables were assessed with a two-tailed *t*-test or *χ*^2^ analysis. The effect of clinical variables on patient survival was analyzed using Cox proportional hazards regression analyses, and Kaplan-Meier survival analysis was used to compare CRC patient survival with the log-rank test. For correlation, we used Spearman correlation. *p* < 0.05 was considered statistically significant.

## Electronic supplementary material

Supplementary figure legend(DOCX 11 kb)

Supplementary Figure 1(JPG 1392 kb)

Supplementary Excel 1(XLSX 12 kb)

Supplementary Excel 2(XLSX 29 kb)

Supplementary Excel 3(XLSX 12 kb)
