# Supplementary material for: TRIP13 promotes tumor growth and is associated with poor prognosis in colorectal cancer
Source: Cell Death Dis. 2018 Mar 14;9(3):402. doi: 10.1038/s41419-018-0434-z (PMC5852242; doi:10.1038/s41419-018-0434-z)
Supplement: Supplementary file 1 — Supplementary figure legend(DOCX 11 kb) [file 41419_2018_434_MOESM1_ESM.docx]

Figure S1. Suppression of TRIP13 by LV10-shTRIP13 inhibits the oncogenic phenotype in vitro and regulates EMT.

(A) Confirmation of the efficiency of TRIP13 knockdown. The upper panel is signal intensity, and the lower panel is the quantified results. The values indicate the mean ± standard deviation.* p<0.05, and **p<0.01.

(B-E) The effects of TRIP13 loss of function on in vitro proliferation (B-C), migration (D), and invasion (E). TRIP13 knockdown inhibits cell proliferation, migration and invasion abilities. The values indicate the mean ± standard deviation. *p<0.05, **p<0.01, and ***p<0.001.

(F) Western blotting was performed in HCT116 and SW480 cells to determine the change in EMT marker expression upon loss function of TRIP13. The signal intensity and quantitative analysis are as shown. The values indicate the mean ± standard deviation.
