# Supplementary figures and images for: TRIP13 promotes tumor growth and is associated with poor prognosis in colorectal cancer
Source: Cell Death Dis. 2018 Mar 14;9(3):402. doi: 10.1038/s41419-018-0434-z (PMC5852242; doi:10.1038/s41419-018-0434-z)

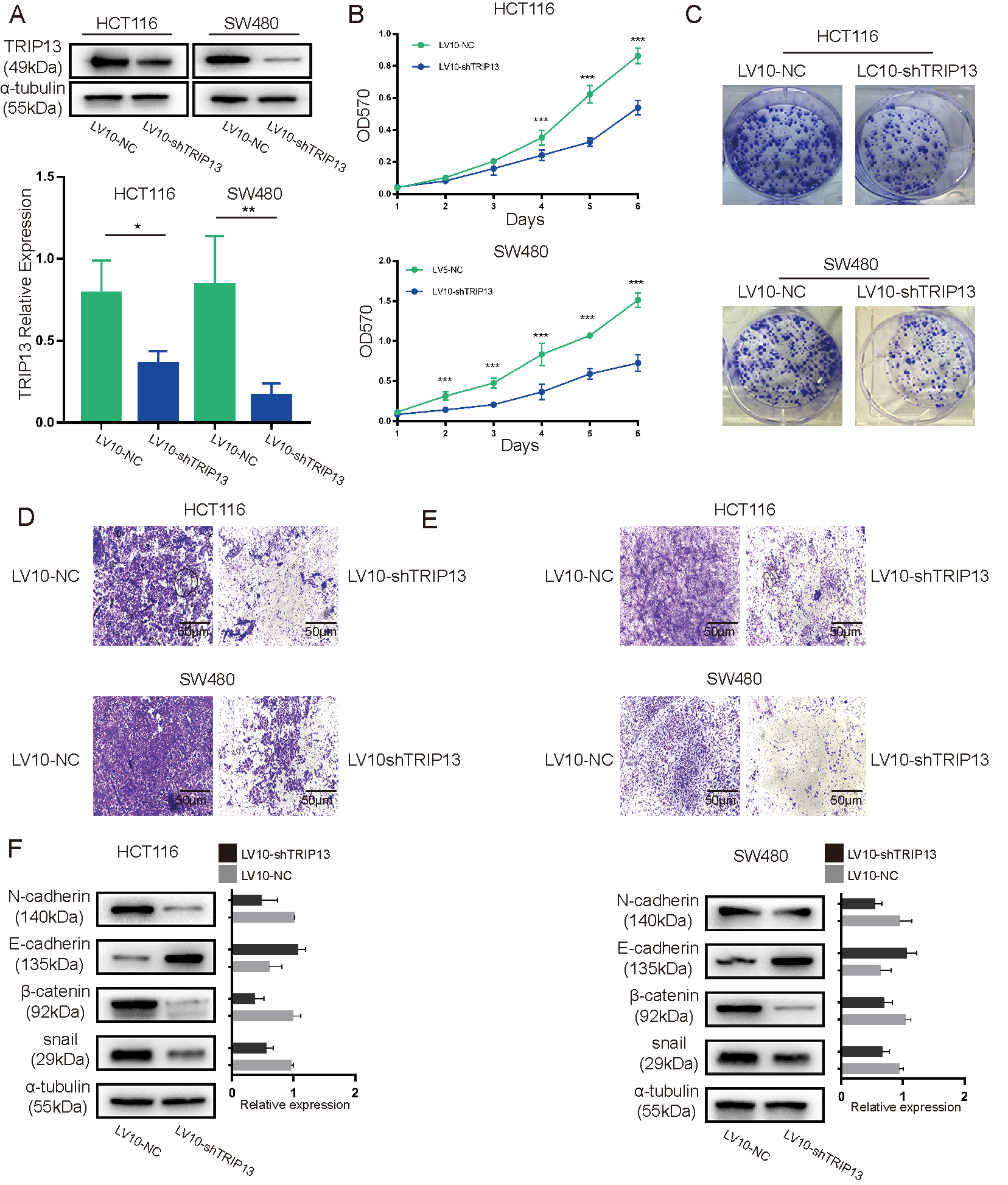

Supplement: Supplementary file 2 — Supplementary Figure 1(JPG 1392 kb) [file 41419_2018_434_MOESM2_ESM.jpg]
